# Waking up of sleeping giant ligno-cellulosic biomass for biofuel production

**DOI:** 10.1093/sumbio/qvag008

**Published:** 2026-03-24

**Authors:** Digambar V Gokhale

**Affiliations:** CSIR—National Chemical Laboratory, Pune 411008, India

## Sustainability statement

The study highlights the production of second-generation bioethanol from ligno-cellulosic biomass (LCB), which does not hinder the food chain supply required for the human consumption, thus avoiding the food versus fuel debates. LCB is abundantly available, which is rich in cellulose, hemicellulose, and lignin requiring advanced processing to convert into fuel (SDG12). LCB conversion to ethanol offers a renewable energy alternative to nonrenewable fossil fuels, thus reducing the dependence on fossil fuels (SDG7). Biofuels like ethanol or butanol not only reduce the negative effect of fossil fuels but also mitigate greenhouse gas emissions, thus fighting with the climate change.

The world population and industrial development rely heavily on fossil fuels to meet their energy demands. The higher consumption of fossil fuels leads not only to their scarcity and sustainability challenges but also release greenhouse gases creating environmental problems. Many nations are committed to zero greenhouse gas emissions by 2050 and hence it is really essential to explore sustainable alternatives to fossil fuels (Oloyede et al. 2024). Diverting food-based resources to biofuel production is not attractive because it leads to imbalance in the food and feed supply chain and also sustainability issues. These concerns have spurred significant research and investment into developing second-generation biofuels from non-food-based sources like LCB. LCB includes agricultural residues, energy crops, municipal solid waste, and wood by-products whose traditional management methods are inefficient to exploit their potential. LCB has enormous potential as sustainable feedstock for fuel and chemical production (Ingle et al. 2025). Typically, LCB is a complex matrix consisting of cellulose (35%–55%), hemicellulose (20%–35%), and lignin (10%–25%). These components are predominantly found in secondary cell walls of plant and are intricately cross-linked to give structural integrity to the plant. Cellulose and hemicellulose are interconnected with each other by hydrogen bonds and covalent bonds and both are linked to lignin forming a complex matrix, which is tough and highly recalcitrant to enzymatic attack (Tamo et al. 2025). Bio-refinery concept was originated during late 1990s along with the birth of green chemistry. It involves the processing of plant biomass into fuels and chemicals using either extraction or conversion processes. Bio-refineries use renewable feedstock and hence are considered sustainable alternatives to oil refineries. They mimic oil refineries by extracting valuable products from a single feedstock, leading to versatile bio-based economy. Integration of bio-refinery processes for LCB valorization minimizes the waste streams and boost the economic value of LCB by efficiently converting all components into biofuels and chemicals (Pandey et al. 2025, Woiciechowski et al. 2025). Many products such as fuels, polymers, and solvents are synthesized from LCB in the new generations of bio-refineries, but real challenge is the cost of bio-based products, which still exceeds the cost of petrochemical products. Hence, it is necessary to improve LCB transformation technologies to make them economically competent during the scaled-up operations (Calvo-Flores and Martin-Martinez 2022). During LCB processing in pulping industries and bio-refineries, the focus is mainly on valorization of carbohydrate (cellulose and hemicellulose) with much less importance given to lignin valorization. Lignin stream (about 99%) is presently burnt to generate heat and electricity and only a small portion (1% or 2%) is valorized commercially. New strategy known as “lignin-first” has been developed, which provides homogeneous lignin suitable for conversion to value-added products through chemical or biological routes. The lignin-first approach enables complete utilization of LCB components, which may possibly lead to economically and environmentally benign processes for producing biofuels and chemicals (Luo et al. 2023, Martin et al. 2024).

Production of ethanol from any LCB source is a multistep process, which involves four core steps: sustainable supply, pretreatment, enzymatic hydrolysis, and fermentation and ethanol recovery. The sustainable supply of LCB is the main logistical challenge, which includes harvesting, collection, transportation, and storage of LCB. Biomass transport affects the economics of biomass processing due to limiting infrastructure (roads) and biomass handling (unloading docks). In addition, long-term storage may result in loss of dry matter, leading to lower ethanol yields (Singhvi and Gokhale 2019). Pretreatment is necessary to break down the recalcitrance plant structure followed by converting carbohydrates (cellulose and hemicellulose) into sugars using lingo-cellulosic degrading enzymes. The sugars released after hydrolysis are converted by fermentation to ethanol which is recovered by distillation. The recalcitrance nature of LCB has to be brought into a reactive state via pretreatment, which improves the accessibility of its components. The pretreatment disrupts the intramolecular and intermolecular hydrogen bonds between cellulose, hemicellulose, and lignin, which assists in the dissolution of biomass. It enhances the permeability and surface area of the biomass and also reduces the cellulose crystallinity so that it becomes easier for enzymes to degrade cellulose into fermentable sugars. Several pretreatment methods have been reported in the literature, which include wet oxidation, steam explosion, ionic liquid, organosolv, physical/mechanical pretreatments, acid, and alkali. Several reviews have been published on LCB pretreatments, highlighting the advantages and disadvantages of the individual method (Kululo et al. 2025, Woźniak et al. 2025). Though physical methods are less invasive, they pose scale-up challenges due to the requirement of high energy. The acids (sulphuric acid or hydrochloric acid) used for pretreatment are considered unsafe due to their poisonous and corrosive properties. In addition, inhibitors such as furfural and hydroxyl-methylfurfural are produced during acid pretreatment, which affect the fermentation process. Alkali pretreatment removes lignin and some hemicellulose, improving the accessibility of biomass to enzymes, resulting in enhancement LCB saccharification. The non-recovery of alkali is the major hurdle that leads to the waste generation and environmental pollution. The use of organic solvents for LCB pretreatment looks promising since it removes lignin in a highly pure form, which can be converted into various high-value products (Nair et al. 2023). However, there are challenges facing in equipment development and solvent recycling, which requires careful management at the industrial scale because solvents are volatile and inflammable. Among the pretreatment methods, deep eutectic solvents (DESs) are found to be effective for LCB pretreatment because they offer green, cost-effective, and efficient lignin removal (60%–100%), increasing the cellulose accessibility. The DESs offer advantages such as recyclability, lower toxicity, and affordability, making them a more sustainable choice for effective LCB pretreatment (Bao et al. 2025; Rajapakse et al. 2025). Recently, microwave-based DES pretreatment resulted in 96% lignin removal from wheat straw just in 150 s at 130°C (Wang et al. 2025), which significantly improved digestibility (92%) of the pretreated wheat straw after enzymatic hydrolysis with 44% cost reduction. However, DES is not yet commercially applicable for LCB pretreatment due to high solvent viscosity and challenges in scaled-up operations. Thus, LCB pretreatment still remains a major issue in second-generation ethanol production. The ideal LCB pretreatment should minimize inhibitor formation, energy consumption, and environmental impact and also maximize the recovery of fermentable sugars. In addition, “one-size-fits-all” approach is not applicable due to varying compositions of LCB feedstock. Further research should focus on combined approaches in leveraging the strengths of multiple methods to effectively overcome the barrier of biomass recalcitrance.

There are many industrial companies involved in cellulolytic enzyme production. These enzymes find wide applications not only in bio-refinery industry but also in textile, detergents, food, and paper industries. Companies aim at developing cost-effective cellulase enzyme cocktails for efficient LCB degradation. The cocktails include several types of cellulases, hemicellulases, β-glucosidases, and oxidative enzymes like lytic polysaccharide monooxygenases (LPMOs) required for total degradation of LCB to sugars. Novozymes and Genencore (DuPont) are globally known major players specialized in producing commercial cellulase enzyme cocktails. The cellulase enzymes market is about USD 1250 million in 2025 and expected to reach USD 2050 million by 2035 (https://www.factmr.com/report/cellulose-enzyme-market). The cellulase enzyme blends produced by Novozymes are called Cellic^®^, which were used by Crescentino, Italy, in largest ethanol plants launched in 2013 for the production of cellulosic ethanol. The Cellic^®^ enzymes hydrolyse a variety of LCBs such as wheat straw, rice straw, bagasse, corn stover, and cobs. The advantages of using Cellic^®^ are reduction in hydrolysis time, increase in the solid loading, reduction in enzyme doses, and enhancement in biomass to sugar conversion. Cellic^®^CTec is a cellulase mixture containing cellobiohydrolases, β-glucosidase, and endoglucanase. Several improved versions of Cellic^®^CTec have been launched by Novozymes such as Cellic^®^CTec 2 and Cellic^®^CHTec 2. These improved versions are five times better in LCB degradation compared to other enzymes available in the market (Ilic et al. 2023). Cellic®CTec 3, introduced by Novozymes, was designed to enhance LCB conversion to sugars. It is a combination of cellulases augmented with LPMOs, improved β-glucosidases, and a new set of hemicellulases. Cellic®CTec 3 HS, another improved version, is a highly efficient cellulase and hemicellulase complex with advanced Auxiliary Activity Family 9 (AA9) LPMOs and improved β-glucosidases. Genencore designed powerful LCB-degrading enzyme cocktails named Accelerase^®^ 1000, Accelerase^®^ 1500, Accelerase^®^ DUET, and Accelerase^®^ TRIO. Among all Accelerase^®^ enzyme cocktails, Accelerase^®^ TRIO offers maximum dose reduction, which, in turn, reduces the ethanol production cost. This enzyme complex contains engineered β-glucosidase in addition to other cellulases, hemicellulases, and LPMOs, leading to reduced product inhibition, thereby increasing the biomass conversion (Ilic et al. 2023). Advances have been made in genetic engineering, metabolic engineering, and protein engineering to boost cellulase production at an affordable cost. However, the overall cost still remains a challenge preventing cellulosic ethanol production from competing with fossil fuels. Researchers are focussing on other multifaceted strategies such as the use of inexpensive substrates, integrated production facilities, enzyme immobilization, and recycling to reduce the cellulase cost. Further technological advances are required to bridge the cost gap and make cellulosic ethanol production competitive with fossil fuels. Raizen, Brazil, inaugurated its second second-generation ethanol plant at Bonfim Bioenergy Park in Guariba, Sao Paulo, in 2024 and the first one is in Costa Pinto Bioenergy Park. Both plants produce 112 million liters of second-generation ethanol per year (https://www.argusmedia.com/en/news-and-insights/latest-market-news/2580045-brazil-s-raizen-ships-2g-ethanol-cargo-to-eu).

The common processes employed for second-generation ethanol production are separate hydrolysis and fermentation (SHF), simultaneous saccharification and fermentation (SSF), simultaneous saccharification and co-fermentation (SSCF), and consolidated bioprocessing (CBP) (Devos and Colla 2022). A recent study showed that higher ethanol production (48.7 g/L) was achieved in SHF compared to SSF (29.5 g/L) from sweet sorghum bagasse. However, SHF is still not commercially attractive due to much longer time taken for ethanol production (Yousefian et al. 2024). SSF integrates both hydrolysis and fermentation processes in one vessel, which improves the economics as well as reduces the residence time during ethanol formation. This approach has been used in ethanol and butanol production from LCB. However, it is very difficult to achieve the optimum temperatures for enzymatic hydrolysis (50°C) and fermentation (30°C–37°C), which, in turn, slows down the ethanol production (Amandio et al. 2023). Another drawback of the SSF process is the inability of wild-type yeasts to ferment xylose efficiently. Researchers have been successful in introducing xylose reductase and xylitol dehydrogenase (XR-XDH) or xylose isomerase (XI) pathway into *Saccharomyces cerevisiae*. These engineered strains can ferment xylose and glucose simultaneously, improving the efficiency of the LCB to ethanol process (Wagner and Gasch 2023). CBP approach is worth exploring for producing biofuels and chemicals from LCB. It integrates cellulolytic enzyme production, LCB hydrolysis, and fermentation of LCB sugars in a single step. Therefore, it is considered an appropriate strategy for the advancement of bio-refinery processes. It uses either one organism or a consortium of microbes to perform enzyme production, LCB hydrolysis, and fermentation in a single vessel initially with the aim of eliminating separate enzymatic hydrolysis, thus reducing the overall bio-refinery process cost (Li et al. 2024). Though CBP reduces the extent of pretreatment compared to conventional processes, some degree of pretreatment is still required for efficient transformation of LCB to bioethanol. Therefore, total elimination of pretreatment still remains a main challenge, which makes the cellulosic ethanol technology commercially unattractive. Thermophilic cellulolytic fungus, *Myceliophthora thermophile*, was metabolically engineered to produce ethanol directly from cellulose by mimicking the aerobic ethanol fermentation of yeast (Crabtree effect) seen in yeast (Zhang et al. 2023a). The engineered fungus produced 52.8 and 39.8 g/L of ethanol directly from cellulose and corn cob, respectively, without the need of cellulase supplementation via CBP. This approach of constructing microbes capable of producing their own cellulases is considered a major breakthrough for the CBP process. It eliminates the need for the addition of external costly commercial enzyme cocktails, which makes the LCB-derived ethanol technology cost-effective. Mining of novel microbes capable of degrading untreated LCB is a promising strategy for eliminating/minimizing the harsh and energy-intensive pretreatment processes. This strategy would significantly reduce the costs of second-generation ethanol, making it competitive with petroleum. The conventional yeast, *S. cerevisiae*, engineered for cellulase production is ideal for CBP to convert LCB to ethanol. In addition, it is robust, tolerates ethanol and low pH, and ferments sugars efficiently. Several strategies for expressing cellulase genes in *S. cerevisiae* have been discussed in successfully constructing cellulolytic CBP-enabled yeasts (Fortuin et al. 2025). Among all strategies, CRISPR/Cas9 approach seems to be a promising, versatile, and precise tool in engineering *S. cerevisiae* for cellulase production, which enables the stable integration of multiple cellulase genes in yeast genome. The developed strains secrete core cellobiohydrolases (CBH), endoglucanases (EG), and β-glucosidase (BGL) required for efficient hydrolysis of LCB in CBP systems. The rational metabolic engineering approaches traditionally pinpoint specific genes for improved ethanol production in yeast. This approach faces several limitations due to the complexity of cellular networks and unpredictable systemic effects. For example, introduction of new or modified pathways may disrupt the metabolic balance of the host organism, which may reduce the cell viability. To mitigate these limitations, evolutionary engineering, also known as adaptive laboratory evolution (ALE), seems to be a powerful complementary approach to enhance bioethanol production by yeast. This approach improves sugar utilization and tolerance to inhibitors (furfural, acetic acid) generated through LCB pretreatment, thereby increasing the ethanol yield. It involves random mutation and selection to improve yeast strains for better ethanol production (Topaloglu et al. 2023, Wang et al. 2025). The ALE strategy was used to obtain genetically stable and furfural-resistant *S. cerevisiae* (Yao et al. 2024). The complete genome sequencing and comparative genomic analysis revealed the changes in the genes related to enhanced furfural resistance. The specific strain was constructed using the CRISPR/Cas9 approach to investigate the mechanism of furfural tolerance. Such strains make the entire LCB to ethanol bioconversion process more robust by improving cellulose breakdown in the presence of inhibitors generated through pretreatment. The same (ALE) approach was shown to be effective for increasing thermo-tolerance of cellulolytic *S. cerevisiae*, which enabled 127% enhancement in ethanol production from pretreated wheat straw at 50°C within 24 h (Zhang et al. 2023b).

In conclusion, bio-refinery industry has been pursuing since long development of commercially viable technology for ethanol production from LCB. However, not much success has been achieved due to high production cost and complex processing. Among all the strategies, CBP seems to be an ideal strategy since it simplifies the overall process by integrating three biological steps required for cellulosic ethanol production. In addition, it helps reduce the high cost involved in the SHF, SSF, and SSCF processes such as separate enzyme production, hydrolysis, and fermentation. However, the difficulty in developing a single organism or a consortium performing all these functions is a core challenge, which hinders the commercial viability of the CBP approach for cellulosic ethanol production. Though CBP holds promise, it is still under a developmental stage for its application in biofuel industry. Natural microbial communities are complex and unstable, which limit their use for LCB conversion. Many synthetic tools have been developed recently, which enable to fine-tune these communities for enhanced LCB conversion to biofuels (Troiano and Studer 2025). In spite of pathbreaking discoveries and inventions by researchers, the LCB-based bioethanol technology has reached only up to the demonstration stage. There are challenges in every unit operation starting from sustainable biomass supply, biomass pretreatment, fractionation, saccharification, and conversion of entire biomass to fuels and chemicals. Many biomass-degrading enzyme cocktails are commercially available, but they are not cost-effective and hence the availability of cellulase cocktails at an affordable cost still remains a problem, which impedes the commercial cellulosic ethanol production. The other challenge is the complexity of LCB hydrolysates, which contain both pentoses and hexoses for which the commercialized technologies have not been designed to manage the mixed sugars. The solution to this challenge is to develop microbes capable of converting multiple sugars to ethanol. No doubt research and development efforts over the past decades have substantially reduced the cost of the processes (pretreatment, hydrolysis, and fermentation). However, key challenges still remain, which limit its cost-competitiveness compared to conventional first-generation ethanol.

## Data Availability

No new data were analyzed in support of this article.
